# Feasibility of evaluating the histologic and genetic subtypes of WHO grade II-IV gliomas by diffusion-weighted imaging

**DOI:** 10.1186/s12868-022-00750-8

**Published:** 2022-12-05

**Authors:** Sirui Liu, Yiwei Zhang, Ziren Kong, Chendan Jiang, Yu Wang, Dachun Zhao, Hui You, Wenbin Ma, Feng Feng

**Affiliations:** 1 Department of Radiology, Peking Union Medical College Hospital, Chinese Academy of Medical Sciences and Peking Union Medical College, Beijing, 100730 China; 2 Department of Neurosurgery, Department of Neurology, Peking Union Medical College Hospital, Chinese Academy of Medical Sciences and Peking Union Medical College, Beijing, 100730 China; 3 Department of Pathology, Peking Union Medical College Hospital, Chinese Academy of Medical Sciences and Peking Union Medical College, Beijing, China; 4 Department of Radiology, Shanghai Institute of Medical Imaging, Zhongshan Hospital, Fudan University, Shanghai, China; 5 Department of Radiology, Peking University First Hospital, No.8 Xishiku, Beijing, China

**Keywords:** Diffusion-weighted imaging, Apparent diffusion coefficient, Glioma differentiation, Genetic status

## Abstract

**Background:**

To explore the feasibility of diffusion-weighted imaging (DWI) metrics to predict the histologic subtypes and genetic status of gliomas (e.g., IDH, MGMT, and TERT) noninvasively.

**Methods:**

One hundred and eleven patients with pathologically confirmed WHO grade II-IV gliomas were recruited retrospectively. Apparent diffusion coefficient (ADC) values were measured in solid parts of gliomas on co-registered T2-weighted images and were compared with each other in terms of WHO grading and genotypes using t-tests. Receiver operating characteristic analysis was performed to assess the diagnostic performances of ADC. Subsequently, multiple linear regression was used to find independent variables, which can directly affect ADC values.

**Results:**

The values of overall mean ADC (omADC) and normalized ADC (nADC) of high grade gliomas and IDH wildtype gliomas were lower than low grade gliomas and IDH mutated gliomas (P < 0.05). nADC values showed better diagnostic performance than omADC in identifying tumor grade (AUC: 0.787 vs. 0.750) and IDH status (AUC: 0.836 vs. 0.777). ADC values had limited abilities in distinguishing TERT status (AUC = 0.607 for nADC and 0.617 for omADC) and MGMT status (AUC = 0.651 for nADC). Only tumor grade and IDH status were tightly associated with ADC values.

**Conclusion:**

DWI metrics can predict glioma grading and IDH mutation noninvasively, but have limited use in detecting TERT mutation and MGMT methylation.

## Background

The 2016 World Health Organization (WHO) Classification of brain tumors integrated molecular parameters into histopathologic classification and tumor grading. Extensive analyses have been performed to study the influence of various genetic markers and glioma grading on patients’ survival and treatment with glioma. Among these genetic markers, three are noteworthy because they are common in gliomas and have great values in routine clinical treatment and prognostic prediction. The first one needs to be noted is isocitrate dehydrogenase mutation (IDH-mut), which can define glioma subtype and indicate good prognosis [1]. The second is O6-methylguanine-DNA methyltransferase promoter methylation (MGMT-m), a favorable independent prognostic biomarker, which can predict glioma patients’ response to temozolomide [1]. The third is telomerase reverse transcriptase promoter mutation (TERT-mut), which associates with a worse prognosis [2] and radiotherapy resistance [3].

Since genetic alterations and WHO grading are related to patient management and outcome, it is essential to figure out a useful method, enabling efficient and secure detection of those prognostic factors. Although the histopathologic examination is the gold standard to test genetic markers in glioma, brain surgery and autopsy are risky. Moreover, it is unable to obtain tumor samples from patients without surgical indications. Molecular detection using tumor tissue is too time-consuming to guide treatment before, during, and after operations timely.

Diffusion-weighted imaging (DWI) is a non-invasive method, which has been widely used in the diagnosis of brain tumors. The apparent diffusion coefficient (ADC) values generated from DWI can quantitatively evaluate the cellularity of tissue and movement of water molecules in vivo [4]. Conventional structural magnetic resonance imaging (MRI) features, including glioma location, glioma volume, necrosis, invasiveness, enhancement pattern, and peritumoral edema, have been used to predict the IDH, MGMT, and TERT status [5–8], however with controversies. Recently, MRI-based radiomic signatures have shown the possibility of predicting genotypes of gliomas [9, 10], while the time-consuming methodology has limited its use in routine clinical work. Advanced MRI, including arterial spin labeling imaging (ASL) [11], DWI [12–15], and dynamic susceptibility contrast perfusion imaging (DSC) [2], is used to assess MGMT and TERT status in patients with glioblastomas. However, few studies evaluate the feasibility of ADC in predicting TERT and MGMT status in WHO grade II-IV gliomas. Simultaneously, the result of several studies that correlated ADC values with WHO grading is still controversial [16–18]. Besides, how the glioma grading and genotypes impact the ADC values of gliomas remains unknown.

Therefore, this study aimed to firstly investigate the association between WHO grade and the ADC values of gliomas, secondly evaluate the predictive capability of ADC in genetic markers (e.g., IDH, MGMT, and TERT) in gliomas, thirdly confirm the parameters that affect the ADC values.

## Methods

### Clinical data and groupings

This retrospective study was approved by the Institutional Review Board of Peking Union Medical College Hospital. The requirement for informed consent from patients was waived. A total of 111 adult patients (mean age: 44.3 ± 12.1 years old) were enrolled in this study. They were pathologically diagnosed with primary WHO grade II-IV gliomas between August 2010 and March 2018 at Peking Union Medical College Hospital. Patients who underwent radiotherapy, chemotherapy, or invasive procedures before magnetic resonance imaging (MRI) acquisitions were excluded from this study. The details about the main clinical features, pathological diagnosis, and genetic status of the enrolled patients are listed in Table 1.

**Table 1:** Patient characteristics and genetic types of WHO II-IV gliomas

Characteristics	LGGs		HGGs	Total	P value
	WHO II n = 36 (32.43%)	WHO III n = 32 (28.83%)	WHO IV n = 43 (38.74%)	WHO II-IV n = 111 (100.00%)	LGGs vs HGGs
Age	42.4 $\pm 11.6$	46.5 $\pm 12.4$	57.8 $\pm 15.0$	44.3 $\pm 12.1$	P < 0.0001
Sex					P = 0.035
Male	24 (66.67%)	19 (59.38%)	15 (34.88%)	58 (52.25%)	
Female	12 (33.33%)	13 (40.62%)	28 (65.12%)	53 (47.75%)	
Genetic type					
IDH					P < 0.0001
Mutation	27 (75.00%)	15 (46.88%)	3 (6.98%)	45 (40.54%)	
Wildtype	9 (25.00%)	17 (53.12%)	39 (90.70%)	65 (58.56%)	
NA	0 (0.00%)	0 (0.00%)	1 (2.32%)	1 (0.90%)	
MGMT					P = 0.002
Methylated	28 (77.78%)	21 (65.63%)	17 (39.53%)	66 (59.46%)	
Unmethylated	6 (16.67%)	10 (31.25%)	26 (60.47%)	42 (37.84%)	
NA	2 (5.55%)	1 (3.12%)	0 (0.00%)	3 (2.70%)	
TERT					P = 0.471
Mutation	17 (47.22%)	13 (40.62%)	25 (58.14%)	55 (49.55%)	
Wildtype	17 (47.22%)	15 (46.88%)	13 (30.23%)	45 (40.54%)	
NA	2 (5.56%)	4 (12.50%)	5 (11.63%)	11 (9.91%)	

Unless otherwise noted, data in the table are presented as n (%) or mean $\pm$ standard deviation; NA: not available

*LGGs* low grade gliomas; *HGGs* high grade gliomas

### MRI data acquisition and imaging processing

MRI studies were performed preoperatively on a 3.0-T MRI scanner (Discovery MR750, GE, US). The MRI protocols included an axial T2-periodically rotated overlapping parallel lines with enhanced reconstruction (T2-PROPELLER) sequence (TR, 12507 ms; TE, 91 ms; TA, 97 s; slice thickness, 6 mm and FOV, 240 × 240 mm^2^) and an axial DWI sequence (TR, 3000 ms; TE, 91 ms; TA, 27 s; slice thickness, 6 mm and b value, 0 and 1000 s/mm^2^).

**Fig. 1:**
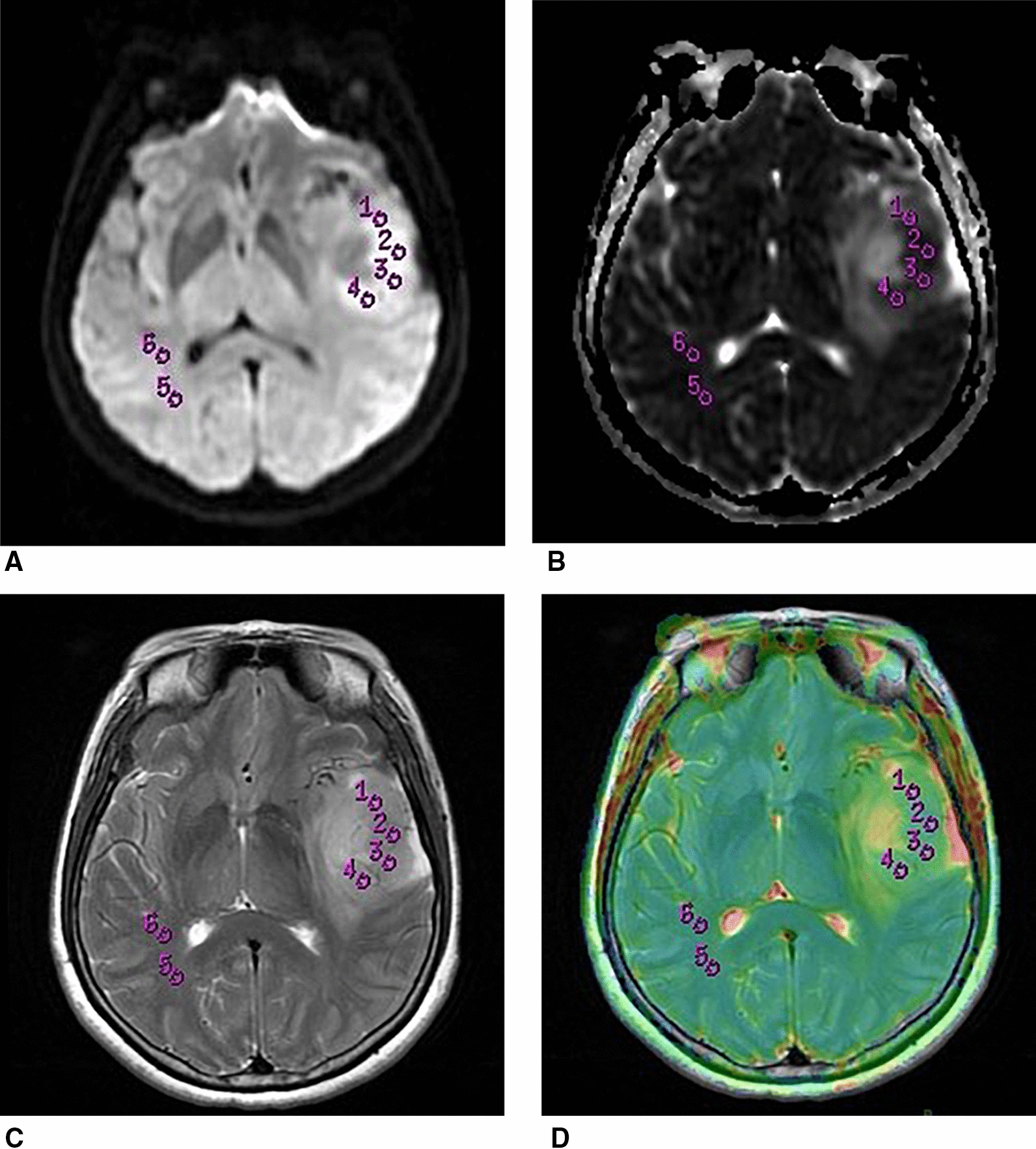
Images showed an example of the placement of ROIs. Axial DWI (**A**), ADC (**B**), T2WI (**C**), and co-registered T2WI (**D**) from a patient with low grade glioma. Four non-overlapping round ROIs were placed within the solid part of the glioma on co-registered T2WI (ROI 1–4), while two same-sized ROIs (ROI 5–6) were placed within the contralateral white matter to calculate the value of the mean ADC in each ROI. DWI: diffusion-weighted imaging; *ADC* apparent diffusion coefficient; *ROI* region of interest

The DWI images were manually transferred to an offline workstation (Advantage Workstation, AW4.5; GE Medical Systems) supplied by the vendor. GE Functool software was further used to generate ADC maps and automatically calculated the mean ADC value for each region of interest (ROI). The solid parts of all the gliomas were confirmed by a consensus of two radiologists blinded to genetic and pathologic information. For each tumor, four ROIs were manually placed within the solid components on co-registered T2-weighted images. Necrotic, cystic, calcified, and hemorrhage areas of gliomas were avoided. Two other ROIs of each patient were selected on the contralateral normal white matter (CNWM) (Fig. 1). The area of each ROI was between 29 to 31 mm^2^.

The formula of the normalized ADC (nADC) value is listed as follows: $\mathrm{nADC}=\frac{\mathrm{overall mean ADC }(\mathrm{omADC})}{\mathrm{ mean }ADC}$, where $\mathrm{omADC is the}$ mean value of the four mean ADC values within tumor and $\mathrm{mean ADC}$ is the mean value of the two mean ADC values within CNWM.

### Histopathology

IDH mutational and TERT promoter mutational analysis was performed using direct sequencing described by Horbinski et al. [19] and Chan et al. [20], respectively. MGMT-m was detected by pyrosequencing reported by Reifenberger et al.[21]. DNA extracted from formalin-fixed, paraffin-embedded tumor tissue was used to detect IDH1/2-mut, TERT-mut, and MGMT-m.

### Statistics

The statistical analyses of data were performed using SPSS, version 20. The Kolmogorov–Smirnov test was used to analyze whether age and ADC data were normally distributed. Chi-square tests were performed to test distribution differences of age, sex, and genetic types between low grade gliomas (LGGs, which refer to WHO grade II gliomas) and HGGs, which refer to WHO grade III-IV gliomas. T-tests were used to compare continuous variables. Statistical significance was set at P < 0.05. Parameters with significant differences were further analyzed by receiver-operating characteristic (ROC) curve to seek the threshold nADC and omADC values to predict genetic status and assess the differentiate performances of nADC and omADC. Multiple linear regression analysis was further performed to test the association of each variable with omADC and nADC.

## Results

### Patient characteristics and genetic type

The detailed baseline characteristics of the 111 patients are shown in Table 1. 36 (32.43%) WHO grade II gliomas, 32 (28.83%) WHO grade III gliomas, and 43 (38.74%) WHO grade IV gliomas were enrolled in this study. IDH and TERT genotype were mutant in 45 (40.54%) and 55 (49.55%) of the 111 gliomas. Gliomas with and without MGMT promoter methylation accounted for 59.46% (66) and 37.84% (42) of all the gliomas, respectively.

Significant differences existed in age (P < 0.0001) and sex (P = 0.035) between LGGs and HGGs (Table 1). Patients in the HGGs group were significantly older than those in the LGGs group. IDH (P < 0.0001) and MGMT status (P = 0.002) were also statistically significant, with more patients in the HGGs group falling into the IDH-wildtype (IDH-wt) category and MGMT promoter unmethylation (MGMT-um) category versus the LGGs group. No significant difference in TERT status was observed.

### Correlation of the ADC values with the WHO grade

**Fig. 2:**
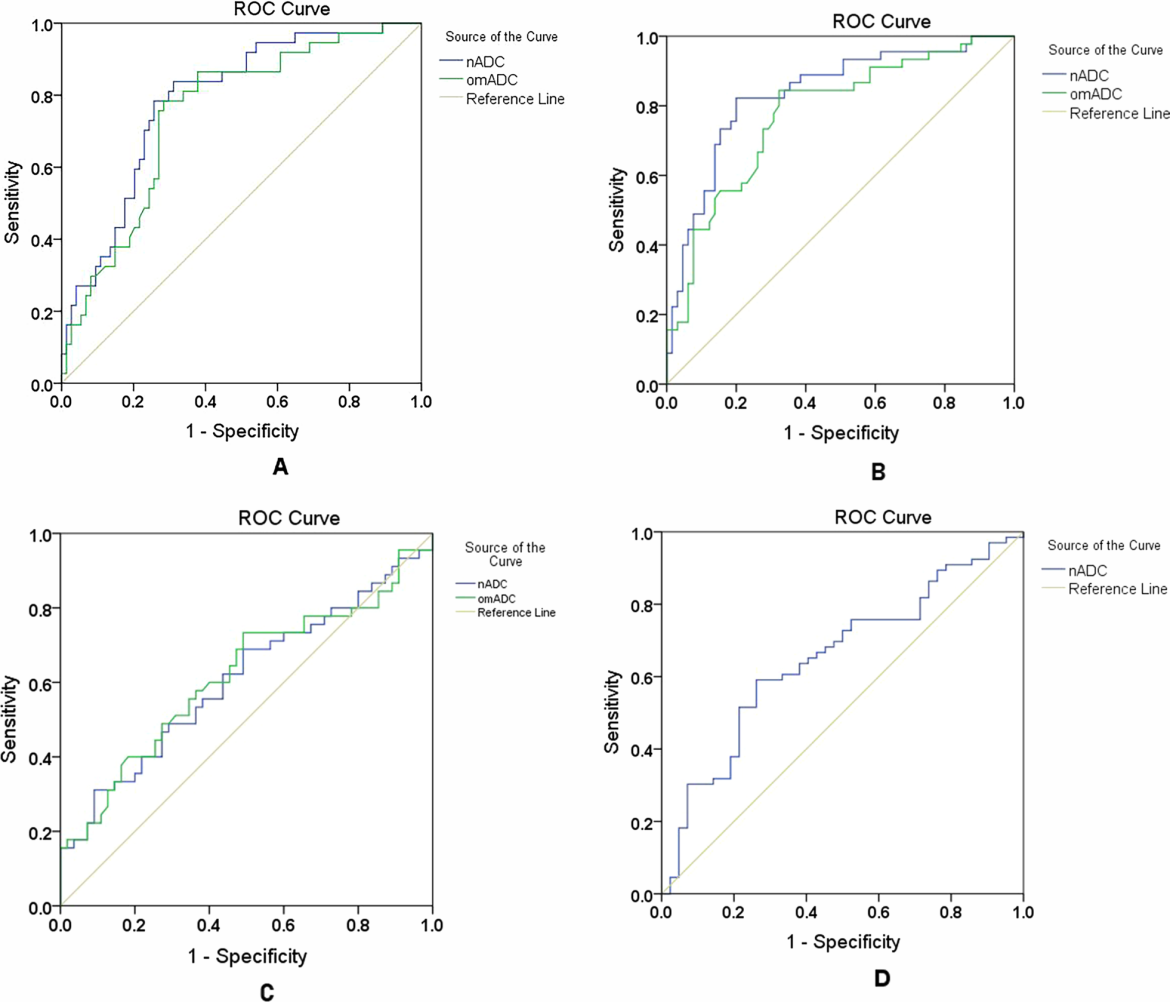
Diagnostic performance of ADC values in WHO-based glioma grading and genotypes. AUC of ROC curves for discrimination between LGGs and HGGs (**A**), IDH-mut and IDH-wt (**B**), TERT-mut and TERT-wt (**C**), as well as between MGMT-m and MGMT-um (**D**), based on nADC and omADC values. *ROC*: receiver-operating characteristic; *LGGs*: low grade gliomas; *HGGs*: high grade gliomas

The values of nADC and omADC were significantly different between LGGs and HGGs according to WHO classification of 2007 (both P < 0.0001). ADC values in LGGs group were higher than those in HGGs group (1.83 ± 0.35 vs. 1.47 ± 0.31 for nADC, and 0.0014 ± 0.0003 vs. 0.0011 ± 0.0003 mm^2^/s for omADC) (Table 2). In ROC analysis, the best cutoff values for nADC and omADC to differentiate LGGs from HGGs were 1.56 (AUC: 0.787, sensitivity and specificity: 83.8% and 68.9%) and 0.0012 mm^2^/s (AUC: 0.750, sensitivity and specificity: 78.4% and 71.6%), respectively (Table 3 and Fig. 2A).

**Table 2:** Summary of discriminant analyses

		nADC (median ± SD)	P value	omADC (median ± SD)	P value
Grade	Low	1.70 ± 0.36	< 0.0001^a^	0.0014 ± 0.0003	< 0.0001^a^
	High	1.42 ± 0.30		0.0011 ± 0.0003	
IDH	Mutation	1.83 ± 0.34	< 0.0001^a^	0.0014 ± 0.0003	< 0.0001^a^
	Wildtype	1.42 ± 0.28		0.0011 ± 0.0002	
MGMT	Methylation	1.65 ± 0.37	0.021^a^	0.0013 ± 0.0003	0.084
	Unmethylation	1.49 ± 0.35		0.0012 ± 0.0003	
TERT	Mutation	1.53 ± 0.27	0.046^a^	0.0012 ± 0.0002	0.041^a^
	Wildtype	1.69 ± 0.44		0.0013 ± 0.0003	

Unit of omADC: mm^2^/s

^a^Significant at p < 0.05; this difference was significant

**Table 3:** Performances of ADC in the comparison of tumor grading and genotypes

	Low grade vs High grade	IDH-mut vs IDH-wt	MGMT-m vs MGMT-um	TERT-mut vs TERT-wt
nADC				
AUC	0.787	0.836	0.651	0.607
95% CI	0.701–0.874	0.757–0.914	0.546–0.757	0.494–0.721
Cutoff value	1.56	1.60	1.59	1.89
Sensitivity	83.8%	82.2%	59.1%	31.0%
Specificity	68.9%	80.0%	73.8%	90.9%
omADC				
AUC	0.750	0.777	NA	0.617
95% CI	0.656–0.844	0.688–0.865		0.503–0.730
Cutoff value	0.0012	0.0012		0.0012
Sensitivity	78.4%	88.4%		73.3%
Specificity	71.6%	67.7%		50.9%

*CI* confidence interval; *Sen* sensitivity; *Sep* specificity; *NA* not available

### Correlation of the ADC values with genotypes

The nADC (1.42 ± 0.28) and omADC (0.0011 ± 0.0002 mm^2^/s) values in IDH-wt gliomas were lower than those (1.83 ± 0.34 and 0.0014 ± 0.0003 mm^2^/s) in IDH-mutated gliomas (both P < 0.0001) (Table 2). In ROC analysis, when the cutoffs were 1.60 and 0.0012 mm^2^/s, respectively, the sensitivities, specificities and AUC of nADC and omADC were 82.2% and 84.4%, 80.0% and 67.7%, and 0.836 and 0.777, respectively (Table 3 and Fig. 2B).

The values of nADC and omADC were higher in TERT promoter wildtype (TERT-wt) gliomas than in TERT-mutated gliomas (P = 0.046 for nADC and 0.041 for omADC) (Table 2). However, these ADC values had limited ability in discriminating TERT status (AUC = 0.607 for nADC and 0.617 for omADC) (Table 3 and Fig. 2C).

MGMT-methylated gliomas exhibited significantly higher nADC values than MGMT-unmethylated gliomas (P = 0.021) (Table 2). However, the predictive performance of nADC was not good (AUC = 0.651, specificity = 73.8%, and sensitivity = 59.1%) (Table 3 and Fig. 2D). MGMT-m could not be detected by omADC values.

### Multiple linear regression analysis of the correlation between the basic information of gliomas and ADC values

Multiple linear regression analysis, including four variables (IDH, MGMT, TERT, and WHO grade), showed that the nADC and omADC values were not statistically affected by TERT status and MGMT status. The values of nADC and omADC were significantly associated with IDH status, and nADC values were also tightly associated with the WHO grade (Table 4). However, only 60.0% of the variation in the nADC values could be explained by WHO grade and IDH status. IDH status exhibited higher nADC values of standardized coefficients than WHO grade (0.311 vs. -0.240) (Table 4), indicating that IDH status has a greater impact on nADC values than WHO grade. The analysis also revealed the trend that lower tumor grade and IDH-mutation status can increase nADC values. The students’ t-tests also demonstrated this trend.

**Table 4:** Results of multiple linear regression analysis

Variables	nADC				omADC			
	B	P value	SE	SC	B	P value	SE	SC
Constant	1.710	< 0.0001	0.098		0.001	< 0.0001	< 0.0001	
Grade	− 0.204	0.011^a^	0.079	− 0.299	< 0.0001	0.053	< 0.0001	− 0.213
IDH-mut	0.311	< 0.0001^a^	0.085	0.380	< 0.0001	0.003^a^	< 0.0001	0.374
MGMT-um	− 0.071	0.340	0.074	− 0.061	− 6.373E-005	0.306	< 0.0001	− 0.107
TERT-mut	− 0.101	0.112	0.063	− 0.167	− 8.526E-005	0.109	< 0.0001	− 0.146
Adjusted R^2^	0.600				0.522			

*SE* standardized error, *SC* standardized coefficients, *IDH-mut* IDH mutation, *MGMT-um* MGMT unmethylation, *TERT-mut* TERT mutation

^a^Significant at p < 0.05; this difference was significant

## Discussion

The 2016 WHO classification of glioma emphasized the role of genetic parameters in glioma patients’ prognosis and treatment response [22]. The identification of histology and genetic status of gliomas before surgery can benefit these patients. DWI is performed as a routine preoperative method for evaluating gliomas. In this case, ADC’s discriminative abilities in histologic subtypes, IDH, MGMT, and TERT status were assessed, respectively.

In the current study, ADC values generated from DWI (b = 0 and 1000 s/mm^2^) decreased significantly with the WHO glioma grade, which was in accordance with previous studies [16, 23]. Cell density, mitotic activity, and vascularity play important roles in gliomas’ pathological grading [24]. For example, the increment of cell density can remarkably restrict water molecules’ movement, which can be reflected by ADC [24]. Therefore, HGGs were more prone to exhibit lower ADC values than LGGs. Louis et al. [25]discovered that HGGs also had lesser normal brain cells and more tumor cells than LGGs, which may also partly explain the lower ADC values in HGGs.

Accurate identification of IDH status is crucial because the prognosis varies greatly according to IDH status. IDH-mutated gliomas have a significantly better prognosis than IDH-wt gliomas [1]. In this study, the IDH-mut rate was 75.00% in LGGs and 24.32% in HGGs, respectively. The IDH-mut rate of HGGs was higher than the reported indices (75% for LGGs and 12% for HGGs) [26]. The ADC values for IDH-mutated gliomas were significantly higher than those for IDH-wt gliomas, which was consistent with previous research [16, 27]. This difference was more significant when high b-value (b = 3000 s/mm^2^) rather than standard b-value (1000 s/mm^2^) was used[23]. IDH may inhibit tumor growth by decreasing the level of nicotinamide adenine dinucleotide phosphate production [26] and hypoxia-inducible factor 1α [28]. This mechanism could decrease cell density and partially explain how IDH-mutated gliomas displayed higher ADC values. Besides, we found that IDH-mut had a direct and greater impact on ADC values than tumor grade, which helped to explain why IDH status could predict prognosis better than the histologic classification [29].Besides IDH, MGMT and TERT are also important genetic hallmarks in guiding clinical treatment and evaluating glioma patients’ prognosis [30, 31]. The ADC values are used as a potential marker for predicting MGMT and TERT status in glioblastomas; however, without expert consensus [2, 12–14, 32]. For WHO II-IV gliomas, we found that ADC values had less accuracy and reliability in discriminating MGMT and TERT status, which limited the use of DWI metrics in predicting these two genotypes. Multiple linear regression analysis also revealed that MGMT and TERT status were not independent parameters for ADC values. We hypothesized that coexisting factors or interactions between variables might induce the increment of ADC in TERT-wt and MGMT-m gliomas. For example, in this study, HGGs were more likely to have MGMT-um and IDH-wt than LGGs (P = 0.002 and P < 0.0001, respectively), and consequently, the ADC values in MGMT-unmethylated gliomas might be affected by the tumor grading and concurrent IDH-wt. In accordance with our results, no significant relationship between ADC values in glioblastomas and TERT [2, 15] and MGMT status [32] was reported. However, several studies [12–14] showed that ADC values were significantly higher in glioblastomas with MGMT-m than with MGMT-um. These conflicting results may be partly due to the difference in ROI selection and subject recruitment [14]. Unlike previous studies [2, 12–15] that only included glioblastomas, this study recruited patients with WHO II-IV gliomas. Besides, we placed ROIs on the solid part of the tumor, which is different from the previous study where ROIs were placed on the contrast-enhanced part of the tumor [25]. The predictive value of ADC values still needs to be verified by further large-scale comparative studies.

Advances in radiomics [9, 10] and MRI techniques, including ASL [11, 16, 33, 34], DSC [2, 33, 35], and diffusion tensor imaging [36, 37], have been used in evaluating glioma grade or genotypes. Several studies [14, 16, 34] have shown that, compared with perfusion parameters, ADC values have a better predictive effect on tumor grade and genotypes. In this study, only ADC values were assessed because DWI is a commonly used sequence and can be performed in all hospitals. Besides, the postprocessing method of DWI is simple and time-saving.

This study’s strength was that it evaluated the discriminative ability of ADC values in WHO glioma grade and various genetic status in the same study. Therefore, an overall assessment of the predictive power of DWI metrics was available. Accessing various genetic features in one study also helped us identify the valuable genotypes which directly affected ADC values. Since higher ADC values were associated with a more favorable prognosis [12, 38], it was crucial to find out meaningful genotypes that were tightly associated with patients’ outcomes.

Besides the intrinsic limitations of retrospective researches, the other four limitations of this study should be noted. Firstly, biopsy samples used in this study were not acquired by ADC-guided biopsy. Because the ROI-based method cannot assess the direct correlation between histopathology and ADC values, some bias can be produced, especially in more heterogeneous gliomas like HGGs. Secondly, the ROIs did not include peri-tumor areas that may also be infiltrated by glioma cells and contain information reflecting tumor genotypes. Thirdly, the sample size was small. Thus, a larger cohort of patients is needed to verify our conclusions. Fourthly, the genotypes evaluated in this study were limited.

## Conclusion

DWI metrics, including nADC and omADC from the solid part of the glioma, have a potential ability to predict tumor grade and IDH-mut, but have limited use in the prediction of TERT-mut and MGMT-m.

## Data Availability

The datasets used and/or analyzed during this study are available from the corresponding author on reasonable request.

## Abbreviations

DWI: Diffusion-weighted imaging
IDH-mut: Isocitrate dehydrogenase mutation
IDH-wt: Isocitrate dehydrogenase wildtype
MGMT-m: O6-methylguanine-DNA methyltransferase promoter methylation
MGMT-um: O6-methylguanine-DNA methyltransferase promoter unmethylation
TERT-mut: Telomerase reverse transcriptase promoter mutation
TERT-wt: Telomerase reverse transcriptase promoter wildtype
ADC: Apparent diffusion coefficient
omADC: Overall mean apparent diffusion coefficient
nADC: Normalized apparent diffusion coefficient
WHO: World Health Organization
T2-PROPELLER: T2-periodically rotated overlapping parallel lines with enhanced reconstruction
ROI: Region of interest
ROC: Receiver-operating characteristic
CNWM: Contralateral normal white matter
HGGs: High grade gliomas
LGGs: Low grade gliomas
